# A smoking cessation intervention for people with severe mental illness treated in ambulatory mental health care (KISMET): study protocol of a randomised controlled trial

**DOI:** 10.1186/s12888-023-04599-x

**Published:** 2023-02-16

**Authors:** Müge H. Küçükaksu, Berno van Meijel, Lola Jansen, Trynke Hoekstra, Marcel C. Adriaanse

**Affiliations:** 1 Department of Health Sciences and Amsterdam Public Health research institute, Faculty of Science, Vrije Universiteit Amsterdam, Van der Boechorststraat 7, 1081BT, Amsterdam, The Netherlands; 2 Department of Psychiatry, Amsterdam UMC and Amsterdam Public Health research institute, Amsterdam, Netherlands; 3 Department of Health, Sports & Welfare, Inholland University of Applied Sciences, De Boelelaan 1109, 1081HV Amsterdam, The Netherlands; 4 Parnassia Psychiatric Institute, The Hague, Netherlands

**Keywords:** Severe mental illness, Smoking cessation, Intervention, Pharmacological treatment, Behavioural counselling, Peer support, Randomised controlled trial

## Abstract

**Background:**

Smoking among people with severe mental illness (SMI) is highly prevalent and strongly associated with poor physical health. Currently, evidence-based smoking cessation interventions are scarce and need to be integrated into current mental health care treatment guidelines and clinical practice. Therefore, the present study aims to evaluate the implementation and effectiveness of a smoking cessation intervention in comparison with usual care in people with SMI treated by Flexible Assertive Community Treatment (FACT) teams in the Netherlands.

**Methods:**

A pragmatic, cluster-randomised controlled trial with embedded process evaluation will be conducted. Randomisation will be performed at the level of FACT teams, which will be assigned to the KISMET intervention or a control group (care as usual). The intervention will include pharmacological treatment combined with behavioural counselling and peer support provided by trained mental health care professionals. The intervention was developed using a Delphi study, through which a consensus was reached on the core elements of the intervention. We aim to include a total of 318 people with SMI (aged 18–65 years) who smoke and desire to quit smoking. The primary outcome is smoking status, as verified by carbon monoxide measurements and self-report. The secondary outcomes are depression and anxiety, psychotic symptoms, physical fitness, cardiovascular risks, substance use, quality of life, and health-related self-efficacy at 12 months. Alongside the trial, a qualitative process evaluation will be conducted to evaluate the barriers to and facilitators of its implementation as well as the satisfaction and experiences of both patients and mental health care professionals.

**Discussion:**

The results of the KISMET trial will contribute to the evidence gap of effective smoking cessation interventions for people treated by FACT teams. Moreover, insights will be obtained regarding the implementation process of the intervention in current mental health care. The outcomes should advance the understanding of the interdependence of physical and mental health and the gradual integration of both within the mental health care system.

**Trial registration:**

Netherlands Trial Register, NTR9783. Registered on 18 October 2021.

## Introduction

Smoking is the leading cause of preventable morbidity and mortality. Among people with severe mental illness (SMI), such as psychotic disorders, smoking is highly prevalent and strongly associated with poor physical health [1, 2]. It is implicated in reducing the life expectancy of people with SMI by 15–25 years, primarily due to cardiovascular diseases, cancer, and respiratory diseases [2, 3]. Approximately 50–80% of people with SMI smoke, a rate approximately two to three times higher than that in the general population [4].

Compared with the general population without psychiatric disorders, people with SMI are a vulnerable group that is more prone to becoming addicted to substances such as tobacco [3, 5]. The increased likelihood of tobacco dependence among people with SMI can be explained by several mechanisms, such as genetic risk factors; for example, schizophrenia and smoking behaviour have been found to be related to several shared genetic loci [6, 7]. Another potential mechanism is socioeconomic status (SES), with people of a lower SES being more likely to have psychotic disorders [8] and to smoke [9] compared with people of a higher SES.

The self-medication hypothesis assumes that people with SMI use smoking to alleviate specific symptoms, such as depressed mood and cognitive problems. It is also assumed that smoking counterbalances the side effects of psychopharmaca [10]. However, no clear evidence exists to support these hypotheses, and the consequences of holding beliefs based on them are critical [11]. The assumptions of self-medication and symptom alleviation through smoking advance the notion that an individual’s mental health problems could worsen following smoking cessation; however, this has not been confirmed by existing research. Systematic reviews have demonstrated that mental health does not deteriorate and even improves moderately following smoking cessation [12, 13]. In addition to expecting unfavourable mental health outcomes, many mental health care professionals (MHCPs) lack confidence in the opportunities for their patients’ successful smoking cessation [14]. This not only impedes the collective process of rethinking smoking within mental health care but also has the potential to deprive people with SMI of appropriate support for smoking cessation.

In the treatment of psychiatric patients, the integration of physical and mental health care is a recently prioritised goal. This has led to the formation of a new body of research on ‘lifestyle psychiatry’ [15]. Systematic reviews have provided evidence that the promotion of a healthy lifestyle, including smoking cessation, has a positive impact on both physical and mental health outcomes for patients with SMI [16, 17]. However, while lifestyle interventions are increasingly being effectively and sustainably implemented within mental health care, a consensus exists that the rate remains rather low and should be improved [18].

In the Netherlands, new policy measures have recently been implemented to reduce tobacco use nationwide, including a smoking ban in all Dutch mental health institutions by 2025 [19]. Currently, most of these institutions do not offer sufficient support for smoking cessation, which is partly due to a lack of effective smoking interventions [20]. Research has demonstrated that behavioural support and pharmacotherapy are the most effective treatments for tobacco addiction. Two forms of behavioural support that are commonly used for people with and without SMI are motivational interviewing [21, 22] and cognitive behavioural therapy (CBT) [23, 24]. Combined CBT and pharmacotherapy treatment has been found to be more effective than CBT alone [24–26]. In terms of pharmacotherapy, the use of varenicline, bupropion, and nicotine replacement therapy have all been found to be safe and effective treatments for smoking cessation in people with SMI [27, 28]. Alongside CBT and pharmacotherapy, the addition of peer support can also be valuable in smoking cessation treatment. Several studies have indicated that it can strengthen individuals’ social network and social support [29], which can positively contribute to the success of smoking cessation [30, 31]. This is especially relevant for people with SMI, who tend to have limited social networks [32, 33].

In the Netherlands, people with SMI are predominantly treated by Flexible Assertive Community (FACT) teams. A FACT team consists of a multidisciplinary team of MHCPs who offer ambulatory care. Such teams aim to offer treatment for psychiatric disorders combined with support for personal, functional, and social recovery. Given the long-term support that they provide to patients with SMI, FACT teams have an excellent opportunity to deliver smoking cessation interventions to them. Currently, no evidence-based smoking cessation intervention has been studied in a Dutch mental health care setting. Therefore, this study aims to evaluate the implementation and effectiveness of a smoking cessation intervention compared with usual care in people with SMI treated by FACT teams in the Netherlands (KISMET). The remainder of this paper is organised as follows. The following section presents the methods that will be applied and includes the inclusion- and exclusion criteria, the contents of the intervention, primary and secondary study outcomes and a statistical analysis plan. The final section discusses the strengths and challenges of the study design as well as the authors’ motivation and contribution of the present study to the research field.

## Methods

### Design

The KISMET smoking cessation intervention will be evaluated in a pragmatic, cluster-randomised controlled trial (cluster-RCT) with a follow-up period of 1 year. Data will be collected at baseline and 3, 6, and 12 month follow-up. A qualitative process evaluation will be conducted alongside the trial.

### Setting

The intervention will be implemented within ambulatory mental health care teams in the Netherlands.

### Participants

Participants will be people with SMI who receive treatment from FACT teams. Patients who fulfil the following criteria will be eligible for inclusion:

- Fulfils the criteria of SMI according to Delespaul and the SMI Consensus Group, which are defined as follows [34]: presenting a non-transient (structural or long-term for several years) psychiatric disorder, for which coordinated care from a care network is necessary and which is not in symptomatic remission; presenting severely impaired social and/or occupational functioning which is not in functional remission;
- Aged ≥18 years;
- Current smoker (≥ 5 cigarettes) without a quit attempt in the past month;
- Expresses an interest in stopping / cutting down smoking;
- Willing and able to sign informed consent.

Patients who are pregnant or breastfeeding, have severe cognitive impairments, present a primary diagnosis of substance use disorder (with the exception of cannabis use disorder), or are experiencing an acute psychiatric crisis or acute physical disease at the time of inclusion will not be eligible to participate.

### Randomisation

Randomisation will be performed by institution at the FACT team level. Through this cluster-randomisation, contamination between the experimental and control groups can be avoided [35]. Before participants are included, the participating FACT teams will be randomly assigned to either the smoking cessation intervention (intervention group) or usual care (control group). The randomisation will be performed by an independent statistician, who is not involved in the execution of the study, using a computer-generated list of random numbers. Due to the nature of the intervention and the study design, the blinding of patients and FACT team members will not possible.

### Recruitment

**Fig. 1:**
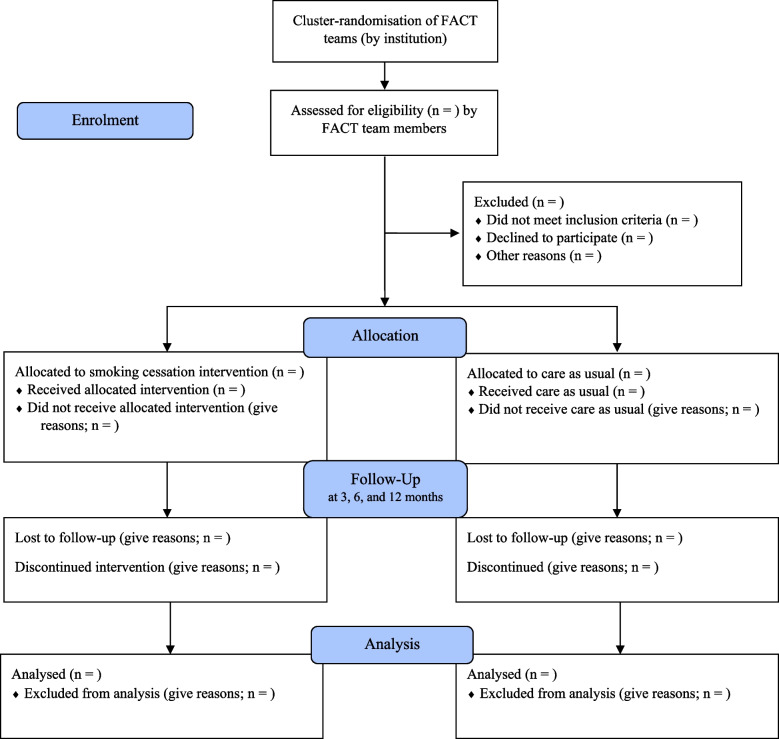
CONSORT participant flow diagram throughout the trial

To recruit eligible patients, the teams will compose a list of possible participants receiving treatment from FACT teams. The coordinating MHCPs will evaluate whether each patient meets the inclusion and exclusion criteria. Eligible patients will be approached by the responsible MHCP and informed about the study, both orally and in writing. The patients will have a minimum of 1 week to decide whether to participate and sign the informed consent form. If a patient decides to participate, he or she will be provided with additional information about the study by the researchers. Figure 1 presents an overview of the study design and participants’ flow throughout the trial:

### Intervention

To develop the intervention, we conducted a three-phase Delphi study. During the Delphi procedures, we established expert opinions on and a critical assessment of the content of the smoking cessation intervention. The finalised KISMET intervention concept entails the following three core components: (1) behavioural counselling (based on the principles of CBT and motivational interviewing); (2) pharmacological treatment (nicotine replacement therapy and other medication); and (3) peer support. Based on the outcomes of the Delphi study, we defined the content and structure of these three core components as well as strategies for optimal implementation, such as frequency, duration, dose, responsibilities of MHCPs, and other organisational aspects. The details of the Delphi study are described elsewhere [36].

#### Core components

1. *Behavioural counselling* will be delivered in groups to create group cohesion and support as well as to reduce the time investment of the FACT teams. Additionally, patients will be offered individual consultations according to their specific needs. They will initially receive psychoeducation about the basic mechanisms of nicotine addiction and what smoking cessation and withdrawal might entail physically and mentally. The information will be comprehensible and facilitated by a personal workbook. An essential part of the psychoeducation will be the normalisation of relapse and lapses to ensure that they are not labelled as failures. This will promote a flexible approach to smoking cessation as a nonlinear process, where (re) lapses are events that can occur in the process of quitting.

These aspects will be addressed in consideration of participants’ psychiatric conditions and their interactions with smoking (cessation). The underlying function and meaning assigned by the patient to smoking will serve as the starting point for finding alternative ways for regulating negative emotions and handling stressful situations *independently.* This notion directly opposes *dependence* on nicotine. Next to learning new coping mechanisms for burdensome emotions, the group sessions will also focus on the influence of smoking on psychotic symptoms, such as hearing voices, and the role of smoking in dealing with these experiences.

The behavioural support will be aimed at helping patients to recognise, accept, and deal with physical, mental, and emotional withdrawal symptoms. Furthermore, personalised relapse prevention plans with self-management techniques will aid in preparing patients for moments in which they anticipate finding themselves vulnerable to relapse.

2. *Pharmacological treatment* is recommended for all patients. The MHCPs will discuss the advantages and disadvantages of pharmacological treatment. The patient and clinician will jointly decide on medication intake based on the patient’s preferences and possible intolerances as well as the clinician’s professional knowledge. The medication choices are nicotine replacement therapy (NRT), varenicline, or bupropion, among which NRT and varenicline are the preferred choices. Doses, possible side effects, and interactions with other medication are well protocoled and based on national and international guidelines [37, 38]. A physician/psychiatrist or clinical nurse specialist will supervise medication use and regularly check in with the patient. Special attention will be paid to patients who take antipsychotic medication, such as Clozapine, as doses are adjusted for people who smoke [39]. Hence, a patient’s plasma levels of Clozapine might rise following smoking cessation, and thus, reducing medication doses by 30% is recommended [40].
3. *Peer support* group meetings will occur on a weekly basis and be facilitated by an expert-by-experience. The expert-by-experience will be a person who has personal experience of addiction, preferably smoking, and will in some cases also have personal experience with SMI. The group meetings will always follow the same structure: first, experiences during past week will be reflected on and exchanged in pairs and plenary, and second, specific topics chosen by the participants will be discussed. The expert-by-experience will play a facilitating and supporting role by sharing their experiential knowledge. The main goal will be to foster patients’ autonomy and self-management.

### Usual care

FACT teams in the control condition will not receive any training and will provide usual care. Thus, people with SMI will have unrestricted access to mental health care and treatment for smoking cessation according to current national guidelines [37]. Furthermore, they will not be allowed to participate in a structured smoking cessation programme for the duration of the study. In case of positive outcomes of this intervention study, control group participants will be offered support to quit smoking in line with the KISMET intervention.

### Training of MHCPs

The KISMET intervention will be provided by trained mental health care workers. Two MHCPs (mental health nurses, clinical nurse specialists, and experts-by-experience) from FACT teams assigned to the intervention condition will participate in a one-day training session. They will function as role models for their colleagues within the FACT team as well as their patients on the subject of smoking cessation and will guide the group sessions. The training will consist of the following: (a) education about pharmacological treatment for smoking cessation, including its effectiveness and possible side and interaction effects; (b) CBT techniques; (c) motivational interviewing techniques; (d) psychoeducation about the connection between SMI, mental health, and tobacco addiction; and (e) principles of peer support. Furthermore, information will be provided about the study design and measurement of the study parameters. The training will be set up and provided by three highly experienced trainers with ample expertise on smoking cessation and mental health. The FACT teams can decide which health care workers will be trained for the intervention. This will promote a greater resemblance to general care as well as enhance the generalisability of the study.

### Main outcome measures

#### Primary outcome

##### Smoking

The primary outcome parameter is smoking behaviour at 12 months. This outcome is defined by exhaled carbon monoxide (CO) measured with a CO monitor and self-report. We define smoking cessation as a CO reading of less than 10 ppm (ppm).

Smoking history and current smoking status will be assessed through a short questionnaire.

##### Nicotine dependence

The degree of nicotine dependence will be measured with the Fagerström Test for Nicotine Dependence (FTND) [41]. This self-report questionnaire contains six items. The *yes/no* items are scored 0 or 1, while the multiple-choice items are scored from 0 to 3. The subscores are added to yield a total score between 0 and 10, indicating low, moderate, or high dependence.

### Secondary outcomes

#### Physical health

##### Body mass index (BMI)

BMI will be computed as follows: bodyweight (kg) divided by the square of height (cm). Body weight will be measured with a scale, while height will be measured with a tape measure.

##### Physical fitness

Physical fitness will be assessed using the 6-minute walk test [42], which was demonstrated to be feasible among people with SMI [43]. In this test, the distance covered by the patient in 6 minutes at a normal walking pace is registered.

##### Systolic and diastolic blood pressure

Blood pressure is defined in mmHg and will be measured twice in a seated position with legs uncrossed after at least 5 minutes of rest. The average of both measurements will be recorded.

##### Lipid profile and glucose metabolism

Serum LDL, HDL, total cholesterol, triglycerides, and fasting plasma glucose metabolism will be measured using venepuncture at the laboratories patients visit for their annual check-ups. All lab measurements will be collected after overnight fasting (8–12 hours). For these measurements, two 7-mL blood samples will be collected (one 4-mL EDTA sample and one 3-mL heparin blood sample).

In case of abnormal blood pressure or blood values, the patient’s general practitioner or coordinating MHCP will be contacted.

#### Patient reports

##### Symptoms of depression and anxiety

Symptoms of depression and anxiety will be measured using the 14-item Hospital Anxiety and Depression Scale (HADS) over the past 4 weeks [44]. This questionnaire consists of 14 items, of which seven items rate anxiety and seven items rate depression. The patient is asked to rate their agreement or disagreement on a 4-point scale. For example, item 13 is ‘I get sudden feelings of panic’ and item 2 is ‘I still enjoy the things I used to enjoy’.

##### Psychotic symptoms

We will assess the severity of psychotic symptoms using the six-item Positive and Negative Syndrome Scales (PANSS-6). The PANSS-6, a shorter version of the original 30-item PANSS, contains only six items and is therefore more feasible [45]. This version is a validated compilation of three negative symptoms (N1 – Blunted affect, N4 – Social withdrawal, and N6 – Lack of spontaneity and flow of conversation) and three positive symptoms (P1 – Delusions, P2 – Conceptual disorganisation, and P3 – Hallucinations). The PANSS-6 will be scored by the MHCP who knows the patient best.

##### Substance use

The use of alcohol, nicotine, and other substances will be measured using the World Health Organization Alcohol, Smoking and Substance Involvement Screening Test (WHO-ASSIST) [46]. This eight-item screening test assesses lifetime and 3-month involvement, dependence, preoccupation with and negative consequences of substance use.

##### Cannabis questionnaire

Cannabis use will be assessed using a self-report questionnaire that contains seven questions regarding frequency, consumption history, quantity, and means of consumption.

##### Health-related self-efficacy

The Patient Activation Measure (PAM-13) is a reliable questionnaire that contains 13 items, which are derived from the original PAM-22 [47]. The questionnaire assesses patients’ self-reported knowledge, skills, and confidence in health-related self-efficacy.

##### Quality of life

Quality of life will be measured using the Short Form-12 (SF-12) questionnaire. The SF-12 is a generic, reliable, and validated instrument that comprises 12 items, which are derived from the SF-36 questionnaire [48]. The physical and mental component summary scores of the SF-12 will be used. Dutch age- and sex-standardised population norms are available [49].

##### Physical activity

Self-reported physical activity will be recorded on each of the four assessments. The frequency per week and impact level of physical activity (low-impact, medium-impact, or strenuous) will be registered.

#### Demographics

Demographic data (i.e., age, gender, educational level, marital status, employment status, diagnosis of SMI, number of years receiving mental health care, and data about past and current medication use) will be collected directly from patient records.

#### Other

*Attendance* at group sessions and peer support group meetings will be registered by the MHCPs delivering the group sessions. *Serious adverse events* will be registered continuously.

Table 1 presents an overview of all outcome measures at each time point:

**Table 1:** Outcome measurements, instruments and data collection schedule

	Baseline	3	6	12
**SMOKING**				
Smoking history	x			x
Current smoking status	x	x	x	x
Number of quit attempts	x			x
Use of electronic cigarettes	x	x	x	x
Use of combustible cigarettes	x	x	x	x
Nicotine Dependence (FTND)	x	x	x	x
Carbon monoxide measurement	x	x	x	x
**PHYSICAL HEALTH**				
BMI (kg/m^2^)	x		x	x
Physical fitness (6-minute walking test)	x			x
Systolic BP (mm/hg)	x		x	x
Diastolic BP (mm/hg)	x		x	x
Lipid profile^b^	x			x
Glucose metabolism (mmol/l)	x			x
**PATIENT REPORTS**				
Symptoms of depression and anxiety (HADS)	x		x	x
Positive and negative symptoms (PANSS-6)	x	x	x	x
Substance Use (WHO-ASSIST)	x		x	x
Cannabis Questionnaire	x		x	x
Health-related self-efficacy (PAM-13)	x		x	x
Quality of life (SF-12)	x		x	x
Self-report physical activity (PA)	x		x	x
**DEMORGAPHICS**^a^				
Age, gender	x			
Education level	x			x
Marital status	x			x
Employment status	x			x
Diagnosis of SMI	x			x
Number of years receiving mental care	x			x
Medication use	x			x
**OTHER**				
Attendance at behavioural support sessions and peer support sessions	Weekly /monthly			
Adverse event reporting	Ongoing collection			

^a^Based on patient records

^b^Cholesterol: HDL, LDL, total and triglyceride (mmol/l)

### Process evaluation

A qualitative process evaluation will be conducted alongside the RCT through semistructured interviews with a selection of FACT team members and patients from the intervention condition. The main objective will be to examine the experiences with and acceptability of the intervention from the perspectives of patients and MHCPs. Furthermore, aspects of the implementation will be evaluated in this process evaluation. At an individual patient level, the aim will be to understand participants’ experiences with and responses to the different elements of the intervention, including the barriers and facilitators experienced when following the KISMET intervention programme. At the level of FACT team professionals, we will seek to understand their experiences and satisfaction with the programme as well as the barriers to and facilitators of the implementation. All interviews will be recorded, transcribed verbatim, pseudonymised, and analysed with the analysis software MAXQDA.

### Sample size

This trial is powered to detect a difference of 15% in smoking abstinence rates between the conditions over 1 year. These rates are expected to be 30% in the intervention group and 15% in the usual care group. This difference is based on the outcomes of multiple previous randomised clinical trials [50]. Based on an expected difference of 15% in smoking cessation rates between the two conditions, the number of participants per group will be *N* = 121. Furthermore, based on results from a previous similar study, we assume a fairly small intraclass correlation coefficient of 0.05. We deem an average cluster size of 10 participants per FACT team to be feasible. We applied a conservative correction factor to account for the multilevel data structure, yielding 12.7 FACT teams. This corresponds to *n* = 127 participants per arm [51]. Assuming a loss to follow-up of 20%, we aim to include a total of 318 participants.

### Statistical analysis

The characteristics of people with SMI in the intervention and control groups will first be presented at baseline and using descriptive statistics (mean [standard deviation], median [range], or frequencies [percentage]). All analyses will be conducted on an intention-to-treat basis, including all randomised patients in the groups to which they were allocated where data are available.

To compare the effects of the intervention with those of usual care on CO-verified smoking cessation (primary outcome) between baseline and 3, 6, and 12 months, a mixed model with a three-level structure (observations clustered within patients, patients clustered within FACT teams, and FACT teams clustered within institutions) will be used, including a random intercept the patient, FACT team, and institution levels [52]. The necessity of random slopes will be assessed using the likelihood ratio test, which will compare the model with random intercepts with a model with random intercepts and random slopes.

The first model will be an ‘overall’ model, with only the treatment variable included. This will provide information about the treatment effect of the primary outcome (odds ratio, 95% confidence interval, and *p*-value), which can be interpreted as the average overall treatment effect over time. Subsequent analyses will include a categorical variable for time (as a dummy variable) as well as an interaction between treatment and time to enable an in-depth investigation of the intervention’s effects at different time points [52].

For binary secondary outcome variables, a similar analysis strategy to the one above will be used. For continuous variables, we will run the first model as mentioned as well as use a longitudinal analysis of covariance to appropriately adjust for the baseline value of the outcome variable [53].

All of the aforementioned models will be additionally adjusted for prognostic variables to increase the precision of the crude treatment effects [54]. These prognostic variables will be identified through a literature study and directed acyclic graphs (DAGs) [55].

Finally, a descriptive analysis will be performed to compare the number of cigarettes smoked per day over time between the two groups as well as attendance at group sessions within the intervention group.

### Handling of missing data

A study demonstrated that mixed models are suitable in situations of longitudinal data with some missing data, in principle averting the need for imputation methods [52]. However, a sensitivity analysis will be run for patients who were allocated to the intervention arm but did not receive any treatment. This sensitivity analysis will include a selective multiple imputation mixed model as described and advised by Twisk et al. [56].

All analyses will be conducted using StataCorp SE version 16.

## Discussion

Smoking among people with SMI is highly prevalent and strongly associated with poor health. Given the high morbidity and mortality rates in individuals with SMI due to diseases directly linked to smoking, an urgent need exists for innovative evidence-based smoking cessation interventions that can be incorporated into mainstream mental health care delivery. This is even more crucial as intervention programmes targeted at the general population do not connect with the needs of people with SMI. This urgent need has been advocated by both researchers and policy makers [57–60] and supported by recommendations from national [61] and international guidelines [62–64]. Moreover, mental health institutions have a great need for effective innovative intervention programmes, including recommendations for successful implementation strategies. Therefore, this study aims to evaluate the implementation and effectiveness of a smoking cessation intervention, compared with usual care, in people with SMI treated by FACT teams in the Netherlands (KISMET).

A strength of this study is the application of a qualitative method (i.e., a Delphi study) to design the intervention before the start of the clinical trial. We established the intervention protocol based on recent scientific findings regarding helpful therapeutic elements for smoking cessation for people with SMI, combined with expert opinions on the contents, structure, and considerations for implementation [36]. Another strength is that a pragmatic study design has been employed, which means that the implementation and evaluation occur in a real-life setting. The outcomes of this study will offer direct insights into the feasibility, acceptability, and effectiveness within current clinical practice, in which we aim to offer the treatment after its effectiveness has been established through this trial. Therefore, the results of this trial will form reliable grounds for formulating clinical implications and recommendations for smoking cessation support in Dutch ambulatory mental health care. An adaptation of this intervention in other countries would possibly require a few adjustments with the consideration of local guidelines for tobacco addiction treatment in the mental health care sector as well as health insurance policies regarding reimbursement for such a treatment. Moreover, the content of the group sessions should be culturally adapted by, for instance, adjusting for distinct attitudes towards smoking.

The recruitment during this trial might pose a challenge. First, recruiting mental health institutions and affiliated FACT teams is difficult, especially in light of the ongoing burden imposed by COVID-19 on the mental health sector and the nationwide introduction of a new funding system. Second, participating FACT teams might encounter difficulties with the inclusion of patients. Recruitment in mental health trials is a common challenge [65]. Therefore, a clear recruitment strategy is essential for including an adequate number of participants. Based on the outcomes of the SCIMITAR trial in similar settings [66], the most critical aspects for successful recruitment are the motivation of MHCPs to support patients in their efforts to quit smoking and their relationship with their patients [67]. A strong therapeutic alliance can positively affect quitting and maintaining abstinence, which is potentially explained by more mutual trust, closer collaboration, and the exchange of positive emotions. These characteristics of a strong therapeutic relationship can facilitate open communication about challenges and difficulties.

Alongside the RCT, we will also conduct a process evaluation at the patient and mental health care professional levels through in-depth interviews. The overall aim is to examine barriers, facilitators, experiences, and perspectives regarding implementation. This approach will combine both quantitative RCT outcomes with qualitative process evaluation outcomes, thereby increasing the internal validity of this study. Thus, we will gain a comprehensive understanding of the working mechanisms of this intervention’s implementation. This will aid in establishing recommendations for further developing the intervention and strategies for (more) effective implementation. Another objective of the process evaluation is to identify relevant aspects for future research as well as how to more effectively promote innovation within the mental health care sector.

Offering a smoking cessation intervention to patients with SMI and training MHCPs to deliver it can empower both patients and professionals. Patients will have the opportunity to learn the required skills and supporting tools for dealing with their tobacco addiction as well as be able to gain more awareness of their own (unhealthy) behavioural patterns. This could increase their self-efficacy and -management of their own physical and mental health. The additional training that the MHCPs will receive could boost their confidence as care providers through increasing their ability to support patients with smoking cessation, thereby improving their somatic and mental health.

Moreover, the experience of successfully implementing a smoking cessation intervention and potentially obtaining positive treatment outcomes could reduce treatment pessimism regarding tobacco addiction. This could in turn alter attitudes based on the stigma that people with SMI are less capable of quitting or do not wish to quit smoking compared with people without SMI.

In light of recent developments, including more attention and efforts allocated to lifestyle promotion within mental health care, we believe that this intervention will contribute to the body of knowledge on ‘lifestyle psychiatry’ and provide insights into the impact of smoking on mental health. We have specifically included secondary outcomes on mental health (i.e., symptoms of anxiety and depression as well as psychotic symptoms) to understand the relationship between smoking, smoking cessation, and mental health and whether it is in line with current literature, which indicates an improvement of mental health after smoking cessation [12].

Overall, this study will provide relevant information on the barriers to and facilitators of the implementation and effectiveness of a smoking cessation programme for people with SMI treated in Dutch ambulatory psychiatric care. If the programme is proven to be feasible and effective, the results will offer a unique venture point for the implementation and its dissemination into ambulatory mental health care. In addition, practical implications will be provided for further policymaking regarding tobacco use as well as future research objectives. These objectives include improving the feasibility and integration of lifestyle-promoting programmes in mental health care. The first results of this trial are expected in 2024.

## Data Availability

Not applicable.

## Abbreviations

BMI: Body mass index
CBT: Cognitive behavioural therapy
CO: Carbon monoxide
FACT: Flexible Assertive Community Treatment
FTND: Fagerström Test for Nicotine Dependence
HADS: Hospital Anxiety and Depression Scale
MHCP: Mental health care professional
PA: Physical activity
PAM-13: Patient Activation Measure
PANSS-6: Positive and Negative Syndrome Scale
RCT: Randomised controlled trial
SF-12: Short-Form Health Survey
SMI: Severe mental illness
WHO-ASSIST: World Health Organization – Alcohol, Smoking and Substance Involvement Screening Test
